# Correction to: Carbon nanotube biocompatibility in plants is determined by their surface chemistry

**DOI:** 10.1186/s12951-022-01302-2

**Published:** 2022-02-17

**Authors:** Eduardo Gonzalez‑Grandio, Gozde S. Demirer, Christopher T. Jackson, Darwin Yang, Sophia Ebert, Kian Molawi, Harald Keller, Markita P. Landry

**Affiliations:** 1 Department of Chemical and Biomolecular Engineering, University of California, Berkeley, CA USA; 2 BASF, Carl-Bosch-Strasse 38, 67056 Ludwigshafen am Rhein, Germany; 3 Innovative Genomics Institute (IGI), Berkeley, CA USA; 4 California Institute for Quantitative Biosciences, QB3, University of California, Berkeley, CA USA; 5 Chan-Zuckerberg Biohub, San Francisco, CA USA; 6 Present Address: Department of Plant Biology and Genome Center, University of California, Davis, CA USA

## Correction to: Journal of Nanobiotechnology (2021) 19:431 10.1186/s12951-021-01178-8

Following publication of the original article [1], the authors identified an error in Fig. 2. The correct Fig. 2 is given in this erratum.

**Fig. 2:**
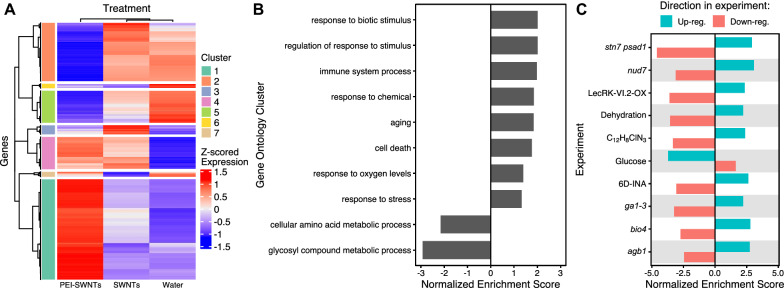
PEI-SWNT responding genes are involved in stress responses, immune system, and programmed cell death. **A** Gene expression heatmap of genes with statistically significant twofold expression change in at least one of the three treatments, compared to non-infiltrated samples. Clusters 1 and 2 show PEI-SWNT specific up- and down-regulated genes. **B**, **C** Gene Set Enrichment Analysis of Cluster 1 and 2 genes using Biological Process Gene Ontology categories (**B**), and Arapath and PlantGSEA databases (**C**). Up- or down-regulation genes in the original experiment were used as independent gene sets to calculate their normalized enriched score. Details corresponding to each experiment can be found in Additional file 4: Table S4C

## References

[1] Gonzalez-Grandio E, Demirer GS, Jackson CT, Yang D, Ebert S, Molawi K, Keller H, Landry MP (2021). Carbon nanotube biocompatibility in plants is determined by their surface chemistry. J Nanobiotechnol.

