# Supplementary material for: Carbon nanotube biocompatibility in plants is determined by their surface chemistry
Source: J Nanobiotechnology. 2021 Dec 20;19:431. doi: 10.1186/s12951-021-01178-8 (PMC8686619; doi:10.1186/s12951-021-01178-8)
Supplement: Supplementary file 1 — Additional file 1: Table S1. Elemental analysis of PEI-SWNTs and source materials used in this study. Mass percentage of each element is reported. Degree of functionalization (DFG) refers to the fraction of carbons on the surface lattice that contain a branched PEI 25 k functional group, calculated as described in the methods section. This DFG represents a 12.54 PEI to SWNT ratio by mass (i.e., 50 ng of PEI-SWNTs contain 627 ng of PEI). Figure S1. Validation of RNA-seq data by RT-qPCR. (A) Normalized log2(RPKM) values for selected genes from Clusters 1 and 2 (PEI-SWNT specific genes) and Clusters 4 and 6 (SWNT specific genes) from RNA-seq data. (B) mRNA levels of same genes as in (A) measured by RT-qPCR. The lower and upper hinges of the boxplot correspond to the first and third quartiles, the upper and lower whiskers correspond to the largest value no further than 1.5 times the inter-quartile range. Statistical significance was determined by a one-way ANOVA with post-hoc Tukey HSD test. Letters denote significant differences among means (n = 5 in A and n = 3 in B). Figure S2. Infiltrated free PEI is more toxic than when conjugated to SWNTs. (A) Arabidopsis leaves infiltrated with water, free PEI (627 ng/µL) and PEI-SWNTs (50 ng/µL) 2-dpi. (B) mRNA levels of the selected marker genes measured by RT-qPCR in leaves of plants infiltrated as in (A). The lower and upper hinges of the boxplot correspond to the first and third quartiles, the upper and lower whiskers correspond to the largest value no further than 1.5 times the inter-quartile range. Statistical significance was determined by a one-way ANOVA with post-hoc Tukey HSD test. Letters denote significant differences among means (n = 3). [file 12951_2021_1178_MOESM1_ESM.pdf]

Sample	%C	%H	%N	DFG
COOH-SWNTs	62.89	2.29	1.49	N.D.
PEI	51.17	11.72	29.50	N.D.
PEI-SWNTs	39.35	8.31	19.87	1/166

Table S1. Elemental analysis of PEI-SWNTs and source materials used in this study. Mass percentage of each element is reported. Degree of functionalization (DFG) refers to the fraction of carbons on the surface lattice that contain a branched PEI 25k functional group, calculated as described in the methods section. This DFG represents a 12.54 PEI to SWNT ratio by mass (i.e., 50 ng of PEI-SWNTs contain 627 ng of PEI).

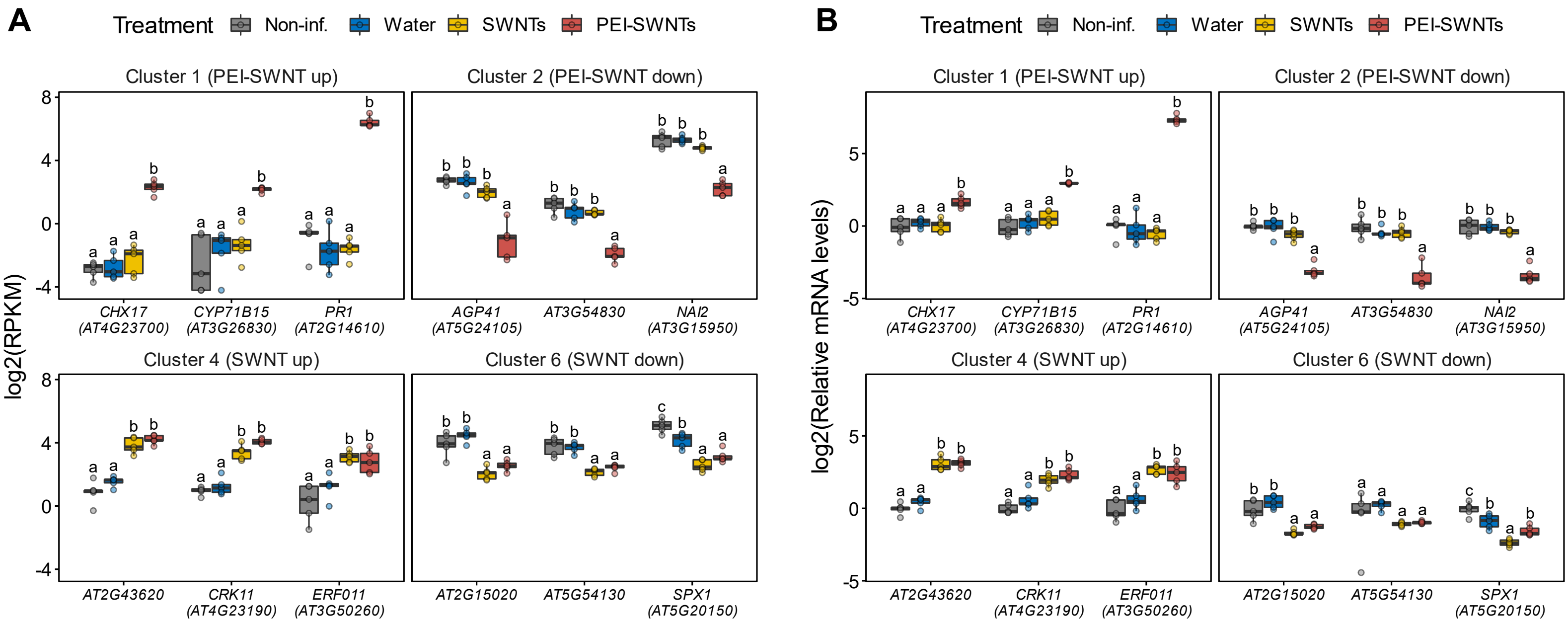

Figure S1. Validation of RNA-seq data by RT-qPCR. (A) Normalized log2(RPKM) values for selected genes from Clusters 1 and 2 (PEI-SWNT specific genes) and Clusters 4 and 6 (SWNT specific genes) from RNA-seq data. (B) mRNA levels of same genes as in (A) measured by RT-qPCR. The lower and upper hinges of the boxplot correspond to the first and third quartiles, the upper and lower whiskers correspond to the largest value no further than 1.5 times the inter-quartile range. Statistical significance was determined by a one-way ANOVA with post-hoc Tukey HSD test. Letters denote significant differences among means (n = 5 in A and n = 3 in B).

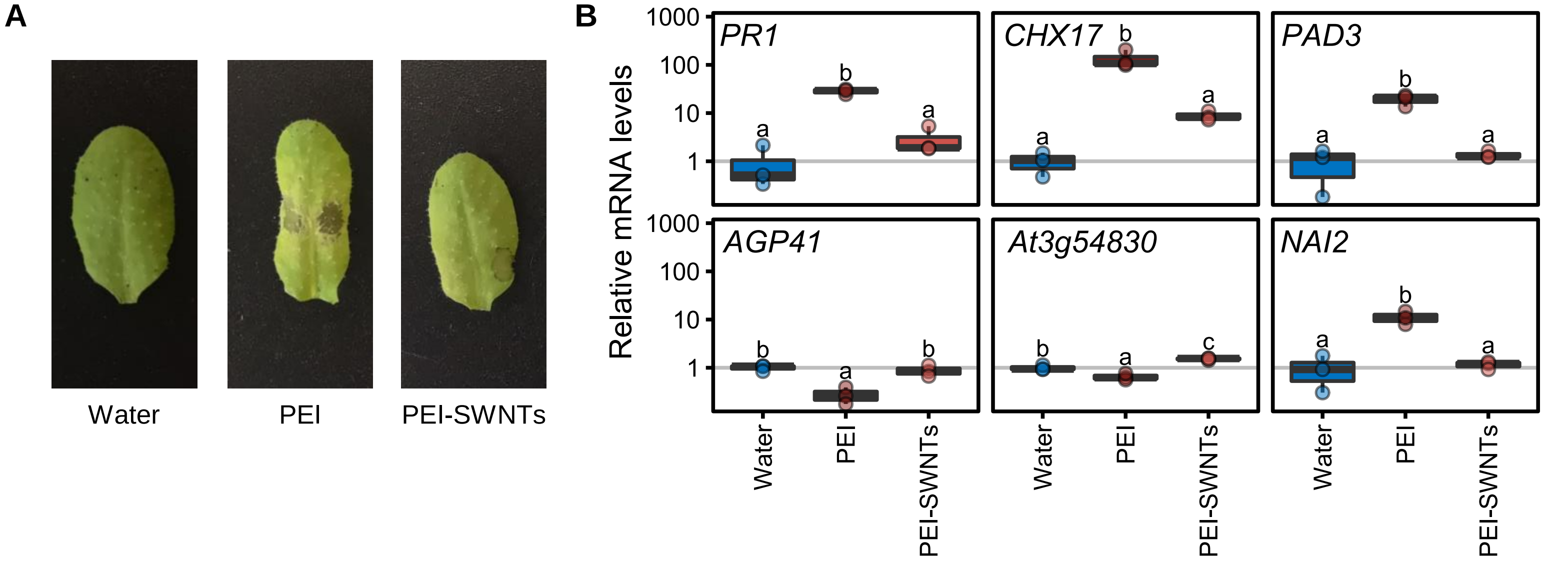

Figure S2. Infiltrated free PEI is more toxic than when conjugated to SWNTs. (A) Arabidopsis leaves infiltrated with water, free PEI (627 ng/µL) and PEI-SWNTs (50 ng/µL) 2-dpi. (B) mRNA levels of the selected marker genes measured by RT-qPCR in leaves of plants infiltrated as in (A). The lower and upper hinges of the boxplot correspond to the first and third quartiles, the upper and lower whiskers correspond to the largest value no further than 1.5 times the inter-quartile range. Statistical significance was determined by a one-way ANOVA with post-hoc Tukey HSD test. Letters denote significant differences among means (n = 3).
